# A Facile One-Pot Synthesis of Au/Cu_2_O Nanocomposites for Nonenzymatic Detection of Hydrogen Peroxide

**DOI:** 10.1186/s11671-015-0935-y

**Published:** 2015-06-03

**Authors:** Ting Chen, Liangliang Tian, Yuan Chen, Bitao Liu, Jin Zhang

**Affiliations:** Research Institute for New Materials Technology, Chongqing University of Arts and Sciences, Yongchuan, Chongqing 402160 China

**Keywords:** Au/Cu_2_O nanocomposites, Nonenzymatic, Hydrogen peroxide detection

## Abstract

Au/Cu_2_O nanocomposites were successfully synthesized by a facile one-pot redox reaction without additional reducing agent under room temperature. The morphologies and structures of the as-prepared products were characterized by scanning electron microscopy (SEM), transmission electron microscopy (TEM), and X-ray diffraction (XRD). The electrocatalytic performance of Au/Cu_2_O nanocomposites towards hydrogen peroxide was evaluated by cyclic voltammetry (CV) and chronoamperometry (CA). The prepared Au/Cu_2_O nanocomposite electrode showed a wide linear range from 25 to 11.2 mM (*R* = 0.9989) with a low detection limit of 1.05 μM (*S*/*N* = 3) and high sensitivity of 292.89 mA mM^−1^ cm^−2^. The enhanced performance for H_2_O_2_ detection can be attributed to the introduction of Au and the synergistic effect between Au and Cu_2_O. It is demonstrated that the Au/Cu_2_O nanocomposites material could be a promising candidate for H_2_O_2_ detection.

## Background

In recent years, the accurate determination of H_2_O_2_ has attracted considerable attentions because it is an important intermediate in various fields, such as food, pharmaceutical, clinical, industrial, and environmental analyses [1–4]. Up to now, a quantity of techniques including spectrometry [5], titrimetry [6], chemiluminescence [7], and electrochemistry [8] have been developed for the quantification of H_2_O_2_. Among the above-mentioned techniques, electrochemical method is attractive due to its low-expense, perfect selectivity, high-sensitivity, and straightforward manipulation [9–11]. Although the enzyme-based electrochemical H_2_O_2_ sensors exhibit obvious advantages of high selectivity, the complicated immobilization procedure, poor stability, and high cost of the enzymes still limit their extensive applications [12, 13]. Thus, the development of enzyme-free H_2_O_2_ sensors with peroxidase-like activity and enhanced performance has become a trend.

Nowadays, numerous materials have been successfully applied to construct nonenzymatic H_2_O_2_ sensors, such as Prussian blue, noble metals, transition metal oxides, carbon nanotubes, graphene, etc [1, 14–19]. It is well known that noble metals were widely used in H_2_O_2_ detection and displayed excellent performance. Particularly, Au nanomaterial exhibits good catalytic activity towards the detection of H_2_O_2_ owing to its outstanding conductivity, electroactivity, biocompatibility and nontoxicity [20]. However, single-phase Au is too unstable to control synthesis, which suffers from difficulties such as the control of particle size, the use of stabilizing agent, and high cost. Hence, it has attracted increasing attention to Au-based nanocomposites. Up to now, Au-graphene nanosheet [21], Au-MnO_2_ [22], and Au-Fe_3_O_4_ [23] have been reported to build H_2_O_2_ sensors. However, the preparation of these materials is a complicated and difficult process. Au nanoparticles were prepared by a few steps in advance and Au-based nanocomposites were obtained using various assistants under specific conditions. It is multistep, time consuming and high cost. Hence, it is essential to develop a green, environmental friendly, low-cost and efficient approach for the synthesis of Au-based nanocomposites.

Cu_2_O is an important semiconductor, which is widely used in solar energy conversion, catalysis, gas sensors, etc [24–26]. The value of Cu_2_O/Cu redox pair is 0.36 V, which is much lower than that of AuCl_4_^−^/Au (0.93 V). Consequently, Au nanoparticles can be obtained decorating onto Cu_2_O through a redox reaction using Cu_2_O as the reducing agent. Moreover, Cu_2_O also has been reported as the electrocatalytic material for H_2_O_2_ detection [27, 28]. Excellent performance can be obtained by the combination of Cu_2_O and Au. In this paper, Au/Cu_2_O nanocomposites have been successfully prepared through a facile, one-pot, and green redox process using Cu_2_O as the reducing agent. Due to the introduction of Au and the synergistic effect between Au and Cu_2_O, the as-prepared product exhibited eminent performance for H_2_O_2_ detection. It is found that the Au/Cu_2_O nanocomposite electrode exhibits high sensitivity and low detection limit towards the reduction of H_2_O_2_. Conclusively, with the straightforward preparation and enhanced performance of Au/Cu_2_O nanocomposite electrode, the Au/Cu_2_O nanocomposite material could be a promising candidate for nonenzymatic H_2_O_2_ sensing.

## Methods

### Chemicals and Materials

Chloroauric acid, dopamine (DA), uric acid (UA), and Nafion solution (5.0 wt% in a mixture of low aliphatic alcohols and water) were purchased from Sigma-Aldrich (St. Louis, MO, USA). H_2_O_2_ (30 wt%), CuCl_2_ · 2H_2_O, NaH_2_PO_4_, Na_2_HPO_4_, ascorbic acid (AA), and glucose (Glu) were purchased from Chengdu Kelong Chemical Reagent (Chengdu, China). All the chemical reagents were of analytical grade and used as received without further purification. Ultrapure water (18.25 MΩ cm^−1^) was used for all experiments.

### Synthesis of Cubic Cu_2_O

In order to prepare cubic Cu_2_O, 10 mL of 2 M NaOH aqueous solution was dropped into the transparent light green CuCl_2_ · 2H_2_O aqueous solution (100 mL, 0.01 M) under vigorous stirring at 55 °C. After stirring for 0.5 h, 10 mL of 0.6 M AA solution was added into the above dark brown turbid liquid and stirred for another 3 h. Finally, the precipitates were collected by centrifugation, followed by washing thoroughly with distilled water and ethanol before freeze drying.

### Synthesis of Au/Cu_2_O

Au/Cu_2_O nanocomposites were prepared via a one-pot, straightforward, and cost-efficient approach. Typically, 15 mg Cu_2_O was dispersed in 10-mL distilled water by ultrasonic dispersion for 10 min, and then, 40-mg sodium citrate was added under constant stirring. About 15 min later, 0.5 mL of 5-mM chloroauric acid was added and the color of the solution turned into brown black immediately, implying the generation of Au nanoparticles. After 20 min, the resultant Au/Cu_2_O nanocomposites were collected by centrifugation, followed by washing carefully with distilled water and ethanol before freeze drying.

### Electrochemical Measurement

The modified electrode was prepared as follows: glassy carbon electrode (GCE, Ф 3) was polished with 0.3- and 0.05-μm alumina powder carefully and rinsed thoroughly with distilled water, followed by sonication in ethanol, nitric acid (1:1), and distilled water, respectively. The prepared nanocomposites were dispersed in 0.5 % Nafion ethanol solution (2 mg/mL) and ultrasonicated for 20 min. Then 5 μL of the suspension was dropped onto the surface of the polished GCE and dried in air.

All electrochemical measurements were performed on a μIII Autolab electrochemical workstation with a standard three-electrode cell in 0.1 M phosphate-buffered solution (PBS, pH = 7.0). Saturated calomel electrode (SCE) and platinum electrode were used as reference electrode and counter electrode, respectively. Cu_2_O-modified GCE (Cu_2_O/GCE) and Au/Cu_2_O-modified GCE (Au/Cu_2_O/GCE) were used as the working electrode. Cyclic voltammetry curves were obtained in the potential range from −0.60 to 0.20 V at different scan rates ranging from 20 to 160 mV/s. Chronoamperometric responses were measured at an applied potential of −0.3 V with the successive injection of different concentration of H_2_O_2_ per 90 s in a constant stirring system.

### Instruments

Morphologies and structures of the prepared products were characterized by field emission scanning electron microscopy (FESEM, Hitachi, SU-8020) equipped with energy dispersion spectroscopy (EDS), transmission electron microscopy (TEM), high-resolution TEM (HRTEM) (FEI-Tecnai G2, USA), and X-ray diffraction (XRD) using Cu-Kα radiation (40 kV, 60 mA).

## Results and Discussion

### Characterizations

**Fig. 1:**
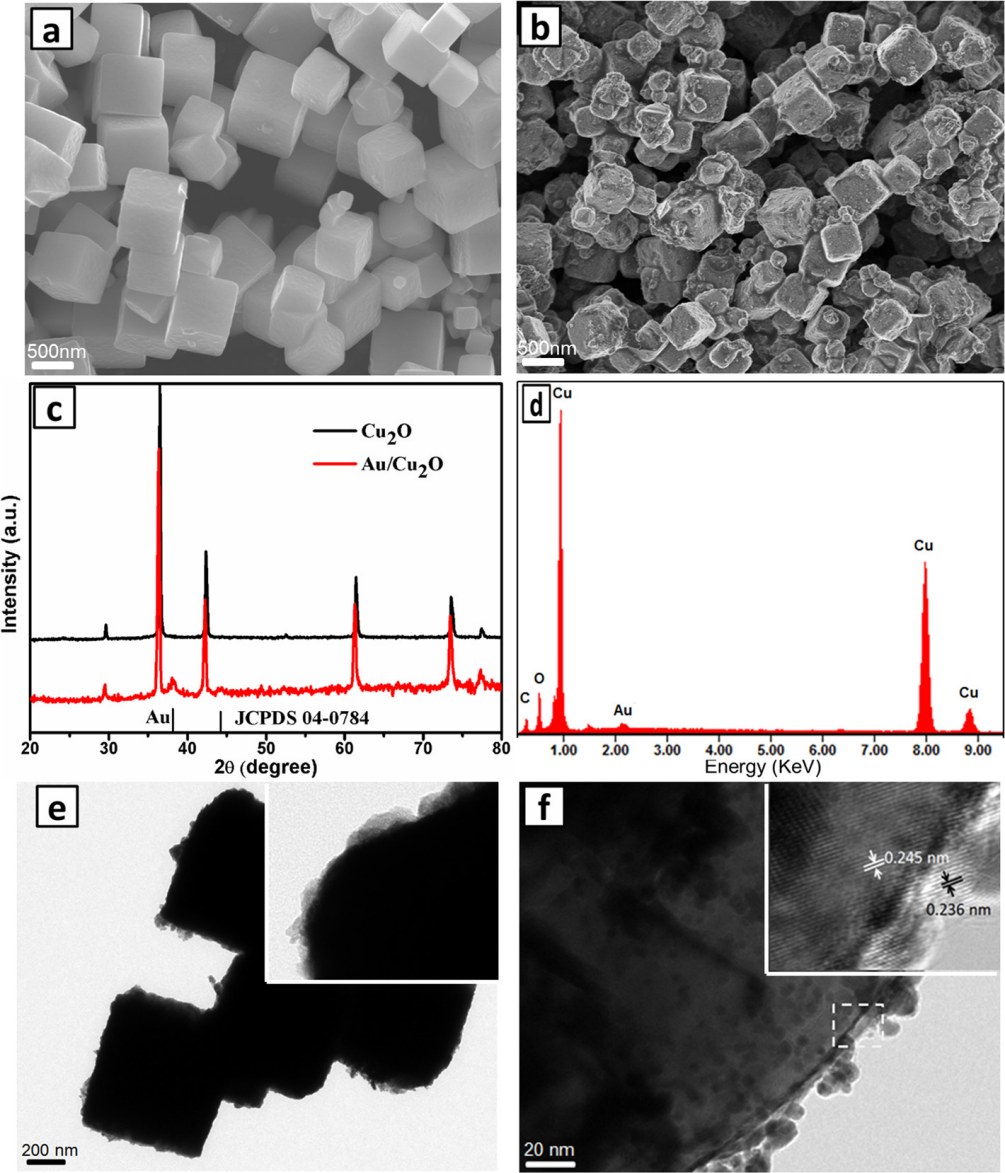
SEM images of Cu_2_O nanocubes (**a**) and Au/Cu_2_O nanocomposites (**b**); XRD patterns of Cu_2_O nanocubes and Au/Cu_2_O nanocomposites (**c**); EDS (**d**), TEM image (**e**), and HRTEM image (**f**) of Au/Cu_2_O nanocomposites

Figure 1a, b displays the SEM images of Cu_2_O nanocubes and Au/Cu_2_O nanocomposites. It is observed that the as-prepared cubic Cu_2_O has smooth surface and relative uniform cubic shape with an average edge length of about 500 nm. Considering the fact that the Cu_2_O/Cu redox pair value is 0.36 V vs. standard hydrogen electrode (SHE), which is much lower than that of AuCl_4_^−^/Au (0.93 V, vs. SHE), therefore, the in situ reduction of AuCl_4_^−^ occurs on the surface of cubic Cu_2_O according to the following redox reaction [29]:

1 $$ 3\mathrm{C}{\mathrm{u}}_2\mathrm{O}+2\mathrm{A}\mathrm{u}\mathrm{C}{{\mathrm{l}}_4}^{-}+6{\mathrm{H}}^{+}=6\mathrm{C}{\mathrm{u}}^{2+}+2\mathrm{A}\mathrm{u}+3{\mathrm{H}}_2\mathrm{O}+8\mathrm{C}{\mathrm{l}}^{-} $$

It is clearly found that the surfaces of the as-prepared Au/Cu_2_O nanocomposites were rough and uneven because of the generation of Au nanoparticles decorated on the surface of Cu_2_O. Figure 1c shows the XRD patterns of Cu_2_O nanocubes and Au/Cu_2_O nanocomposites. All the diffraction peaks of Cu_2_O crystal can be indexed to the standard cuprite structure (JCPDS 05-0667). Compared with the Cu_2_O diffraction pattern, two additional peaks located at about 38.2° and 44.3° were observed, which were assigned to the (111) and (200) diffraction peaks of Au (JCPDS 04-0784). In addition, the EDS spectrum (Fig. 1d) confirms the presence of Au, Cu, and O elements, which agrees with the XRD spectrum analysis. Figure 1e shows the TEM image of Au/Cu_2_O nanocomposites, and it is found that Au/Cu_2_O nanocomposites with defined cubic shapes were distinctly decorated by Au nanoparticles. Furthermore, HRTEM image (Fig. 1f) clearly shows that Au nanoparticles homogeneously distribute on the surface of Cu_2_O cubes and the particle size of Au is about 3 nm. It is observed that the spacing of marked adjacent lattice fringes are about 0.236 and 0.245 nm, which is consistent with the standard value of Au (111) and Cu_2_O (111), respectively. The result is in accordance with the XRD spectrum analysis. The formation of the Au/Cu_2_O heterostructures may be attributed to the similar (111) lattice spacing of Au and Cu_2_O, which forced Au heterogeneous nucleation on the surfaces of Cu_2_O cubes.

### Electrochemical Performance of Au/Cu_2_O/GCE

**Fig. 2:**
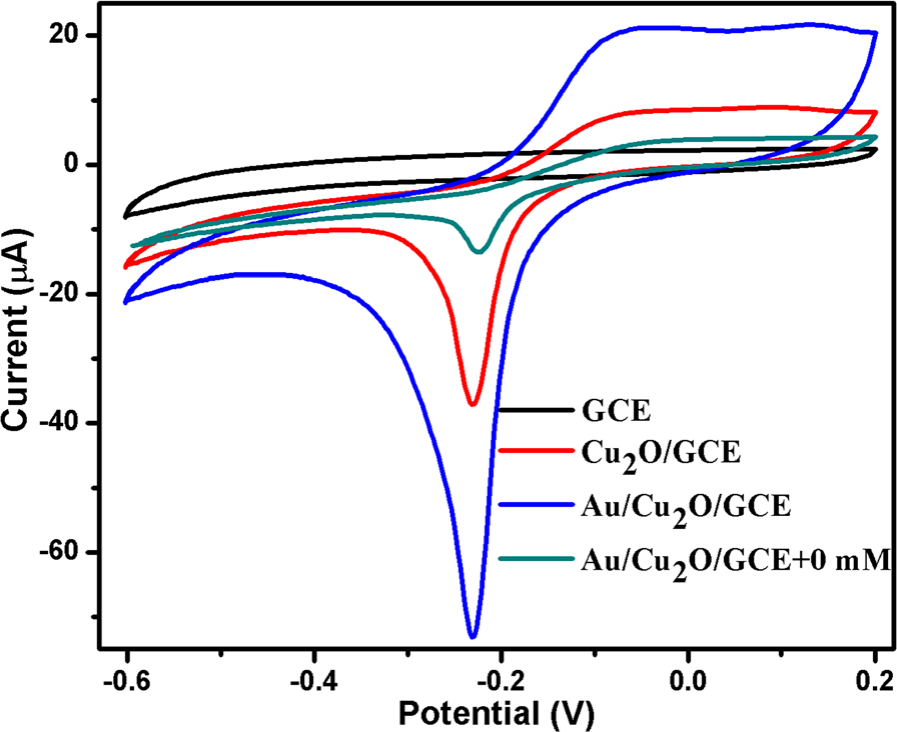
CVs of different electrodes in the presence of 0.5 mM H_2_O_2_ in 0.1 M PBS (pH = 7.0). Scan rate: 100 mV/s

Au/Cu_2_O/GCE was constructed to research the electrochemical performance towards the reduction of H_2_O_2_. Figure 2 shows the electrocatalytic responses of bare GCE, Cu_2_O/GCE, and Au/Cu_2_O/GCE in the presence of 0.5 mM H_2_O_2_ in 0.1 M PBS solution (pH = 7.0). It is found that no reduction peak is observed on bare GCE with the injection of H_2_O_2_ into the PBS solution. Apparently, Cu_2_O/GCE shows a significant reduction peak towards the reduction of H_2_O_2_. It may be ascribed to the fact that Cu^I^ species turned into Cu^III^ species, which is in agreement with the previous reports [30]. Notably, the reduction peak current of H_2_O_2_ on the Au/Cu_2_O/GCE is further increased. It is also important to show that the Au/Cu_2_O/GCE exhibits a quite weak electrochemistry response in the absence of H_2_O_2_. All these observations indicate that Au/Cu_2_O nanocomposites exhibit notable electrocatalytic activity towards the reduction of H_2_O_2_. The excellent activity can be ascribed to the faster electron transfer kinetics, which is caused by the increase of electroactive area and the electron transfer rate. In addition, different work function between Au nanoparticle and Cu_2_O semiconductor leads to the charge redistribution at the interfaces of Au/Cu_2_O nanocomposites [31]. The redistribution of the surface charges may improve the electrocatalytic activity [32]. Comprehensively, the improved catalytic activity for H_2_O_2_ reduction is caused by the introduction of Au and the synergistic effect between Au and Cu_2_O, which is in agreement with the previous reports [22, 23].

**Fig. 3:**
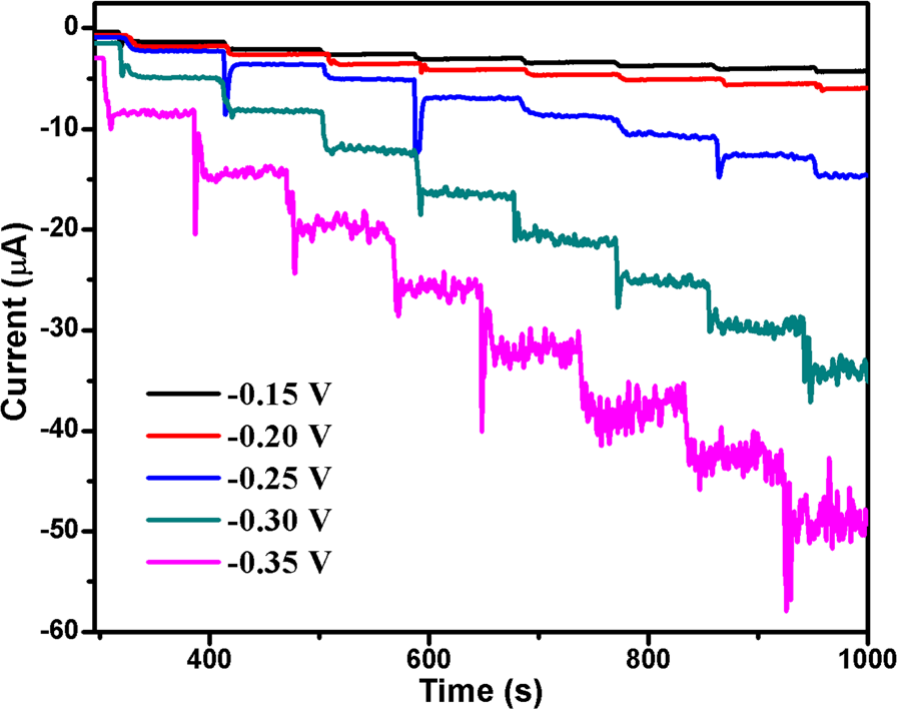
Amperometric current curves of the Au/Cu_2_O/GCE with the successive addition of H_2_O_2_ into the stirring 0.1 M PBS (pH = 7.0) at different potentials

In order to obtain the optimal response to H_2_O_2_, the effect of applied potential on the response current was investigated. Figure 3 shows the amperometric current curves of the Au/Cu_2_O/GCE with the successive addition of 0.1 mM H_2_O_2_ into 0.1 M PBS (pH = 7.0) at different potentials from −0.15 to −0.35 V. It is found that the current response of −0.15, −0.20, and −0.25 V are lower than that of −0.30 and −0.35 V. Compared with the potential of −0.30 V, the response current of −0.35 V is less stable and has larger background noise. In addition, some interfering species which are stable under relatively low potential would be oxidized at high potential [33]. Thus, −0.30 V was chosen as the working potential for the detection of H_2_O_2_.

**Fig. 4:**
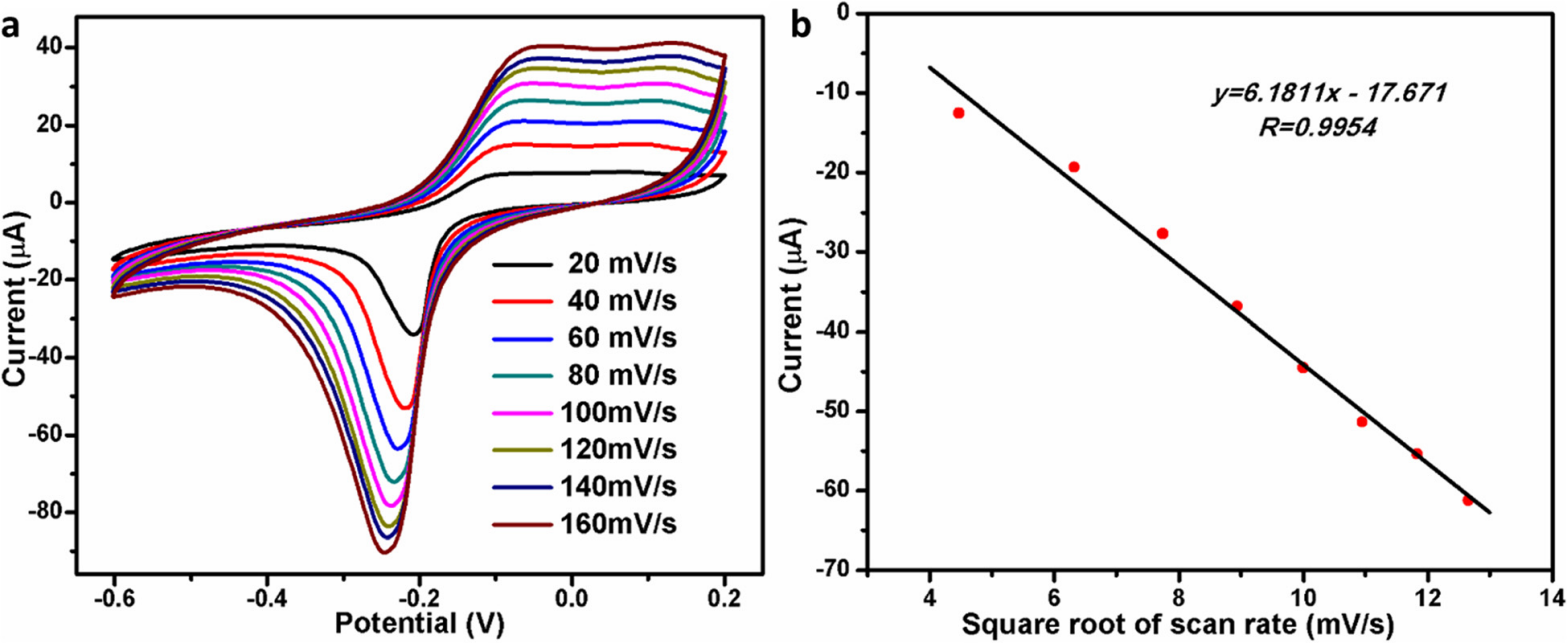
**a** CVs of the Au/Cu_2_O/GCE in the presence of 0.5 mM H_2_O_2_ at different scan rates in 0.1 M PBS (pH = 7.0). **b** The linear relationship between the cathodic current at −0.30 V and the square root of scan rates

**Fig. 5:**
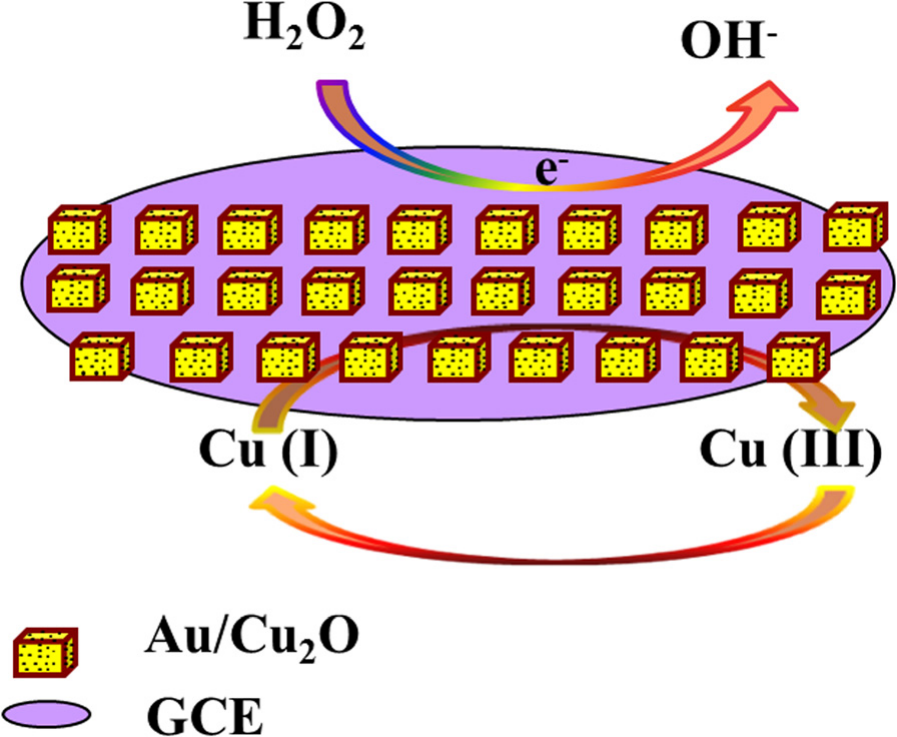
Schematic diagram of the catalytic mechanism on the Au/Cu_2_O/GCE for H_2_O_2_ reduction

To further investigate the electrode kinetic process, the cyclic voltammetries (CVs) of Au/Cu_2_O/GCE at different scan rates were also examined. As shown in Fig. 4, the reduction peak current increases linearly with the square root of the scan rates (*v*^1/2^) in the range of 20 to 160 mV/s (*R* = 0.9954). The result indicates that the electron transfer of Au/Cu_2_O nanocomposites on the GCE is a diffusion-controlled electrochemical process. Comprehensively, a proposed catalytic mechanism for the reduction of H_2_O_2_ on the Au/Cu_2_O/GCE was displayed in Fig. 5. Cu^I^ turned into Cu^III^ species providing electrons to the reduction of H_2_O_2_, and meanwhile, Au accelerated electron transfer kinetics in the electroactive surface area.

### Amperometric Detection of H_2_O_2_ at Au/Cu_2_O/GCE

**Fig. 6:**
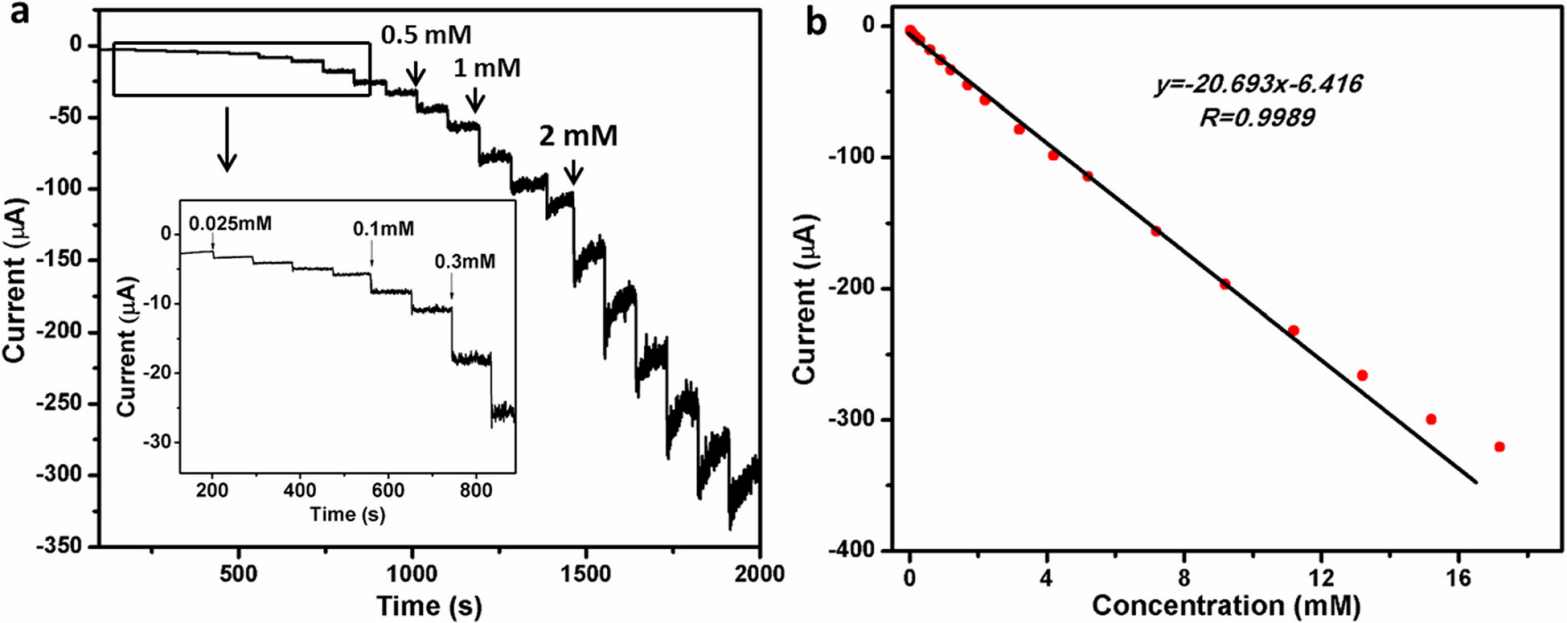
**a** Amperometric current curve of the Au/Cu_2_O/GCE with the successive addition of H_2_O_2_ into the stirring 0.1 M PBS (pH = 7.0) at −0.3 V. Inset is the amplification of the marked rectangle region shown in the curve. **b** The linear relationship between the catalytic current and the concentration of H_2_O_2_

To quantify the electrochemical detection of H_2_O_2_, the Au/Cu_2_O/GCE was evaluated by chronoamperometry (CA). Figure 6a depicts the amperometric current curve of Au/Cu_2_O/GCE at an applied potential of −0.30 V with the successive injection of different concentrations of H_2_O_2_ into PBS (pH = 7.0). The inset of Fig. 6a is a magnified current-time curve at low concentrations. It shows a stepwise increase in agreement with H_2_O_2_ addition and reaches a steady status within 3 s. Figure 6b depicts the linear relationship between catalytic currents and H_2_O_2_ concentrations. The Au/Cu_2_O/GCE for H_2_O_2_ detection shows a wide linear range from 25 μM to 11.2 mM with a lower detection limit of 1.05 μM (*S*/*N* = 3) and high sensitivity of 292.89 μA mM^−1^ cm^−2^. The linear regression equation is *y* = − 20.693*x* − 6.416 [*y*(μA); *x*(mM)] with a correlation coefficient of *R* = 0.9989. Above all, Au/Cu_2_O/GCE exhibited excellent performance towards the reduction of H_2_O_2_. The enhanced electrocatalytic performance could be ascribed to the outstanding conductivity and electroactivity of Au nanoparticles, which accelerates the transfer rate of electrons in the reduction of H_2_O_2_. Table 1 shows the comparison of H_2_O_2_ determination of different modified electrodes. It is shown that Au/Cu_2_O nanocomposites exhibited a wider linear range and lower detection limit towards the detection of H_2_O_2_.

**Table 1:** Comparison of H_2_O_2_ determination of different modified electrodes

	Linear	Limit of	
Electrode	range (μM)	detection (μM)	Reference
Cu_2_O MCs^a^	5–1500	1.5	[34]
Cu_2_O HNs^b^	2–150	1.03	[35]
Cu_2_O/GNs^c^	300–7800	20.8	[36]
Au NPs^d^	100–50,000	4.0	[37]
Ag-Au-rGO	100–5000	1.0	[38]
Au/Cu_2_O NCs^e^	25–11,200	1.05	this work

^a^Cu_2_O microcubes, ^b^Cu_2_O hollow nanocubes, ^c^Cu_2_O nanocubes wrapped by graphene nanosheets, ^d^Au nanoplates, ^e^Au/Cu_2_O nanocomposites

### Interference and Stability Study

**Fig. 7:**
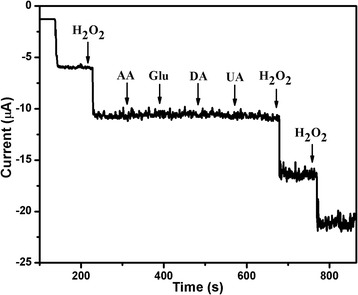
Amperometric current curve of Au/Cu_2_O/GCE with the successive addition of a 0.2 mM concentration of H_2_O_2_, AA, Glu, DA, and UA

To investigate the selectivity of the Au/Cu_2_O/GCE towards H_2_O_2_, several possible interfering species were examined. Figure 7 shows the amperometric response with the successive injection of 0.2 mM H_2_O_2_ and 0.2 mM interfering species, including AA, Glu, DA, and UA at an applied potential of −0.30 V in PBS (pH = 7.0). Apparently, no interference current is found in the testing process indicating the excellent selectivity of Au/Cu_2_O/GCE towards H_2_O_2_. The stability of Au/Cu_2_O/GCE was evaluated by the current step method measuring the amperometric current responses towards 1 mM H_2_O_2_ repeating ten times, and it is found that the relative standard deviation (RSD) was approximately 1.3 %. In addition, the amperometric current response to H_2_O_2_ over a long operational period of 1500 s was about 95 % of its original counterpart. These observations indicate that the Au/Cu_2_O/GCE is relatively stable.

## Conclusions

Au/Cu_2_O nanocomposites were successfully synthesized by a facile one-pot green redox reaction using Cu_2_O as the reducing agent. The Au/Cu_2_O/GCE exhibited excellent performance for nonenzymatic detection of H_2_O_2_ with high selectivity, low detection limit, and strong anti-interference capability. The excellent electrocatalytic activity may be caused by the introduction of Au and the synergistic effect between Au and Cu_2_O. The Au/Cu_2_O nanocomposite material is promising for practical applications in nonenzymatic detection of H_2_O_2_.
